# Cryo-EM Studies of Virus-Antibody Immune Complexes

**DOI:** 10.1007/s12250-019-00190-5

**Published:** 2020-01-08

**Authors:** Na Li, Zhiqiang Li, Yan Fu, Sheng Cao

**Affiliations:** 1 CAS Key Laboratory of Special Pathogens and Biosafety, Wuhan Institute of Virology, Wuhan, 430071 China; 2 Center for Biosafety Mega-Science, Chinese Academy of Sciences, Wuhan, 430071 China; 3 University of Chinese Academy of Sciences, Beijing, 100049 China

**Keywords:** Cryo-electron microscopy (Cryo-EM), Icosahedral, Antigen, Virion, Immune complex

## Abstract

Antibodies play critical roles in neutralizing viral infections and are increasingly used as therapeutic drugs and diagnostic tools. Structural studies on virus-antibody immune complexes are important for better understanding the molecular mechanisms of antibody-mediated neutralization and also provide valuable information for structure-based vaccine design. Cryo-electron microscopy (cryo-EM) has recently matured as a powerful structural technique for studying bio-macromolecular complexes. When combined with X-ray crystallography, cryo-EM provides a routine approach for structurally characterizing the immune complexes formed between icosahedral viruses and their antibodies. In this review, recent advances in the structural understanding of virus-antibody interactions are outlined for whole virions with icosahedral T = pseudo 3 (picornaviruses) and T = 3 (flaviviruses) architectures, focusing on the dynamic nature of viral shells in different functional states. Glycoprotein complexes from pleomorphic enveloped viruses are also discussed as immune complex antigens. Improving our understanding of viral epitope structures using virus-based platforms would provide a fundamental road map for future vaccine development.

## Introduction

Antibodies, the essential components of humoral immunity, are a major defense line against viral infections. To neutralize viral infections, antibodies primarily bind to specific epitopes on the outer surfaces of viral particles. An important first step in modern structural vaccinology involves structurally characterizing the interactions occurring between viral antigens and their cognate antibodies (Anasir and Poh 2019). This step provides the direct evidence for the location of viral epitopes, which helps to elucidate the neutralization mechanisms of antibodies. The first high-resolution structure of a virus (tomato bushy stunt virus) was solved four decades ago using X-ray crystallography (Harrison *et al.*^1978^), a technique that has been primarily limited to determining the structures of relatively simple non-enveloped viruses (http://viperdb.scripps.edu/xray.php) or viral components. Because crystallizing virus-antibody complexes can be challenging, morphological studies of virus-antibody interactions have long been carried out through transmission electron microscopy (TEM) with negative staining protocols (Almeida and Waterson 1969). The resolution achieved by this technique is usually low, but the structural details can be enhanced by modeling high-resolution crystal structures into low-resolution TEM maps. As the negatively-stained samples are visualizable with a conventional TEM instrument, they are still widely used today for characterizing immune complexes with human viruses like influenza A (Ekiert *et al.*^2012^), Marburg (Flyak *et al.*^2015^) and Ebola viruses (Flyak *et al.*^2016^).

Cryo-electron microscopy (cryo-EM), a Nobel-prize-winning technique, is now routinely used for studying virus-antibody complexes at high resolution (Earl and Subramaniam 2016). In contrast to negatively-stained samples, which might be significantly distorted by the dehydration process and the presence of stains, virus particles in cryo-EM studies are freshly frozen in a thin layer of vitreous ice to maintain their native conformations, making high-resolution analyses possible. However, before direct electron detection cameras (DEDs) were introduced in 2012, cryo-EM structures could only be solved at sub-nanometer resolutions with traditional CCD cameras, except some pioneer work based on images recorded on photographic film (Zhang *et al.*^2010^). Largely resulting from the development of DED technologies, better microscopes and sophisticated reconstruction algorithms, cryo-EM has developed in recent years as a powerful high-resolution technique in structural biology research (Shen 2018).

There are two major 3D cryo-EM analysis strategies: single particle analysis (SPA) and cryo-electron tomography (cryo-ET) (Danev *et al.*^2019^). SPA is usually used for macromolecular assemblies that are stable, soluble and homogeneous *in vitro*. Because some viruses are highly structurally ordered, especially those with icosahedral symmetries, it is possible to solve the structures of whole viral particles at better than 3 Å resolution using SPA by combining data from several thousands of purified virus particles (Jiang and Tang 2017). The density maps at this resolution allow the *de novo* building of atomic models. For viruses with pleomorphic shapes, 3D reconstruction of a single vitrified virion is achievable using cryo-ET procedures at ~25-Å resolution. Sub-tomogram averaging techniques are further exploitable for achieving better structural details for repeating structures, such as the surface glycoproteins on viruses. Both SPA and cryo-ET approaches have been used for structural studies on the interactions occurring between whole virions (or viral components) and their associated antibodies.

## Antibodies for Cryo-EM Studies

Antibody molecules for cryo-EM studies can be generated in different ways. Mass production of murine monoclonal antibodies (mAbs) has traditionally been achieved by the use of hybridoma technology where antibody-producing B cells derived from immunized mice are fused to an immortalized cell line (e.g., myeloma) (Kohler and Milstein 1975). Recombinant antibodies can be generated by phage display technology followed by *in vitro* selection, which targets the whole virus particles or some viral components (Hoogenboom 2005). Various methods for isolating antigen-specific human B cells to obtain mAbs have also been reported (Crowe 2017). For viruses with naturally occurring high mutation rates like HIV-1, broadly neutralizing antibodies (bNAbs) that potently target a wide range of viral strains have been isolated from virus-infected individuals and extensively studied using cryo-EM (Stephenson and Barouch 2016; Chuang *et al.*^2019^).

**Fig. 1:**
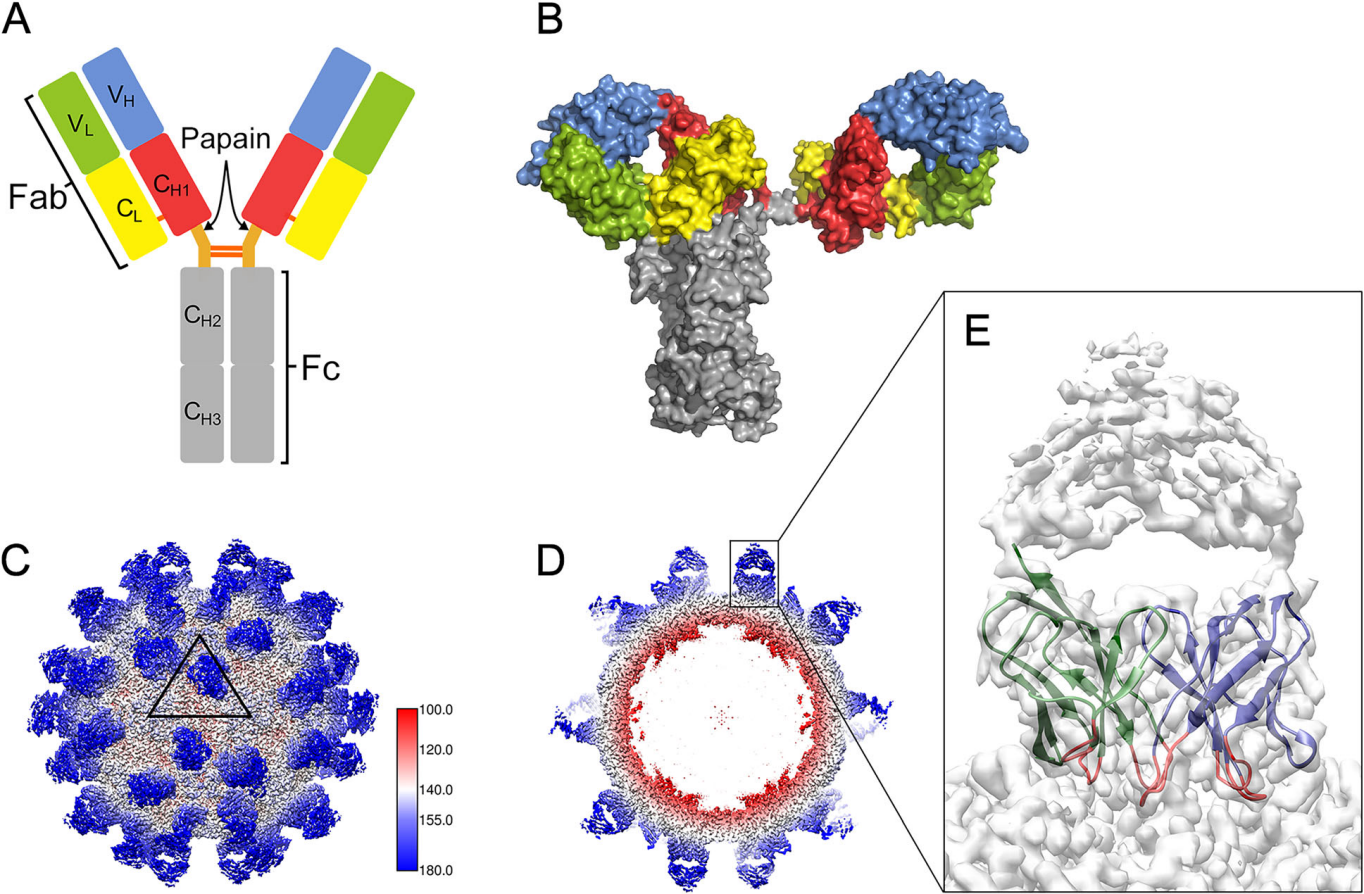
Structural models of antibody molecules on the viral surface. **A** Modular organization of a Y-shaped IgG molecule, which consists of two heavy chains and two light chains covalently linked via disulfide bonds. Papain digestion of a parental antibody produces two Fab fragments and one Fc fragment. A Fab fragment comprises V_H_, C_H1_, V_L_ and C_L_ domains. **B** Surface illustration of highly asymmetric human IgG1 b12 (PDB-1HZH) colored by domain as in (**A**). **C** Cryo-EM map of Fab AT12-015 complexed with HPeV3 (EMD-0069), showing that the 60 copies of Fab (blue) are placed on the viral surface (white). A triangular icosahedral asymmetric unit (ASU) is outlined on the capsid surface. The numbers show the positions of neighboring fivefold and two threefold axes limiting the ASU. **D** Central cross-section of the cryo-EM map showing the Fab densities (blue). **E** The enlarged inset shows the density corresponding to one Fab AT12-015 molecule. The V_H_ and V_L_ domains represented by green and blue ribbons are well defined in the density map. The six complementarity-determining regions in V_H_ and V_L_ are colored red.

For cryo-EM analyses, the antibody components are IgG molecules or their truncated parts. In a single IgG molecule, two antigen binding fragments (Fabs) are present (Fig. 1A, 1B), a situation that could result in highly heterogeneous antigen–antibody complexes if one antigen particle becomes crosslinked with another. Except for SPA studies on IgG bivalency (Ye *et al.*^2016^), IgG is usually used in cryo-ET studies at medium resolution for HIV-1 (Tran *et al.*^2012^), influenza (Tran *et al.*2016b) and Ebola viruses (Tran *et al.*2016a). Compared with the intact IgG molecule, the monovalent Fab fragments generated by papain digestion of whole IgG molecules are more commonly used for structural studies on virus-antibody complexes (Tables 1, 2, 3). The single-chain variable fragment (scFv) generated by fusing the variable regions of the heavy (V_H_) to the light (V_L_) chains with a flexible peptide linker (Finlay *et al.*^2017^) can be used for cryo-EM studies of virus-antibody interactions (Kaufmann *et al.*^2009^; Liu *et al.*^2017^). Single-domain antibodies (sdAbs), such as variable domains of heavy chain-only antibodies (termed VHH) and V_H_ domains of human IgG molecules, have also served as antibody derivatives for cryo-ET studies when combined with sub-tomogram averaging (Meyerson *et al.*^2013^).

**Table 1:** Summary of cryo-EM structures from picornavirus-antibody complexes in the Electron Microscopy Data Bank (EMDB) (https://www.ebi.ac.uk/pdbe/emdb/).

Genus	Virus name	Antigen	Antibody^a^	Fragment	EMD codes	Resolution (Å)
*Enterovirus*	Coxsackievirus (CV)	CV-A6 A-particle	1D5	Fab	6757	3.8
		CV-A10 A-particle	2G8	Fab	9603	4.3
		CV-A10 mature virion	2G8	Fab	9604	3.9
		CV-A10 procapsid	2G8	Fab	9605	4.2
	Enterovirus (EV)	EV-A71 mature virion	MA28-7	Fab	5673	23.4
			E18	Fab	2397	10
			E19	Fab	2436	13
			D5	IgG	6365	7.2
			D5	Fab	6366	4.8
		EV-A71 procapsid	D5	Fab	6383	6
			22A12	Fab	6200	8.8
			E18	Fab	2434	16
			D6	Fab	6963	4.9
			A9	Fab	6964	6.8
		EV-A71 VLP	D5	IgG	6384	5.5
		EV-D68 mature virion	15C5	Fab	9633	3.6
			15C5/11G1	Fab	9634	3.5
		EV-D68 A-particle	11G1	Fab	9636	7.2
	Poliovirus (PV)	PV1 mature virion	A12	Fab	5670	12
			C3	Fab	5291	11.1
			PVSP6A	VHH	5886	4.8
			PVSP29F	VHH	5888	6.5
			PVSS8A	VHH	6433	4.2
			PVSP19B	VHH	6434	4.8
			PVSS21E	VHH	6435	3.8
		PV2 mature virion	A12	Fab	5671	20
		PV1 procapsid	P1	Fab	5283/5284/5285/5286	13/21/18/18
			C3	Fab	5293	22
		PV1 A-particle	P1	Fab	5280/5282	12/26
			C3	Fab	5292	9.1
			PVSP17B	VHH	8285	5.3
			PVSS12B	VHH	8285	5.3
			PVSS10E	VHH	8277	4.8
			PVSS7A	VHH	8286	5.3
	Rhinovirus (RV)	RV B14 mature virion	C5	Fab	8754/8761/8762	2.53/2.71/2.26
		RV B14 procapsid	C5	Fab	8763	3.01
*Aphthovirus*	Foot-and-mouth disease virus (FMDV)	FMDV-O mature virion	D9	Fab	0173	3.97
*Parechovirus*	Human parechovirus (HPeV)	HPeV-3 mature virion	AT12-015	Fab	0069/3138	2.8/15
		HPeV-1 mature virion	AM28	Fab	2761	19.76
*Hepatovirus*	Hepatovirus A (HAV)	HAV mature virion	R10	Fab	6688	4.2
			F4	Fab	9827	3.9
			F6	Fab	9828	3.68
			F7	Fab	9829	3.05
			F9	Fab	9830	3.79

^a^Structural insights into the possible mechanisms for antibody-mediated neutralization discussed in the text are summarized below. 1D5: inhibition of virus-cellular binding (Xu *et al.*^2017^), 2G8: capsid stabilization (Zhu R *et al.*^2018^), MA28-7: cross-linking of virions and blocking receptor binding (Lee *et al.*^2013^), E18: induction of genome release (Plevka *et al.*^2014^), D5: capsid stabilization (Ye *et al.*^2016^), 22A12: capsid stabilization (Shingler *et al.*^2015^), D6: blocking receptor binding (Zhu L *et al.*2018b), A9: blocking receptor binding and capsid destabilization (Zhu L *et al.*2018b), 15C5: blocking receptor binding and locking capsid at intermediate stage, 11G1: locking capsid at intermediate stage (Zheng *et al.*^2019^), R10: blocking receptor binding (Wang X *et al.*^2017^), F4, F6, F7 and F9: blocking receptor binding (Cao *et al.*^2019^).

**Table 2:** Summary of cryo-EM structures from flavivirus-antibody complexes.

Virus name	Antigen	Antibody^a^	Fragment	EMD codes	Resolution (Å)
Dengue virus (DENV)	DENV1 mature virion	14c10	Fab	5268	7
		1F4	Fab	2442	6
	DENV2 mature virion	747(4)B7	Fab	2818	10.24
		1A1D-2	Fab	1418	24
		2D22	Fab	2967/2968/2969/2996/2997/2998/2999	6.5/20/21/6.9/13/11/23
	DENV2 immature virion	2H2	Fab	5674/5675/5676/5677	21/25/21/21
		E53	Fab	5102	23
	DENV3 immature virion	1H10	Fab	9649/9650/9651	12/25/25
	DENV3 mature virion	5J7	Fab	5935	9
West Nile Virus (WNV)	Immature virion	E53	Fab	5103	15
	Mature virion	E16	Fab	1234	14.5
		E16	scFv	5115	22.75
		CR4354	Fab	5190	13.7
Tick-borne encephalitis virus (TBEV)	Mature virion	19/1786	Fab	3754/3755	3.9/19.2
Zika virus (ZIKV)	Mature virion	ZIKV-117	Fab	8548	6.2
		ZKA190	Fab	6793/6794	22/22
		Z23	Fab	9542	9.4
		C10	Fab	9573/9574/9575	4.4/12/4
		ZAb-FLEP	Fab	7613	9.7
		ZK2B10	Fab	9811/9812	20/11
		ZIKV-195	Fab	9131	4
Japanese encephalitis virus (JEV)	Mature virion	2F2	Fab	6854	4.7
		2H4	Fab	6855	4.6

^a^The possible neutralization mechanisms for flavivirus antibodies discussed in the text are summarized below. 14c10: blocking receptor binding (Teoh *et al.*^2012^), 1F4: blocking virus attachment (Fibriansah *et al.*^2014^), 1A1D-2: blocking virus attachment by binding to hidden epitopes (Lok *et al.*^2008^), 2D22: blocking capsid reorganization required for virus fusion (Fibriansah *et al.*2015a), 2H2: inhibition of virus maturation (Wang *et al.*^2013^), E53: binding to partially immature heterogeneous virions (Cherrier *et al.*^2009^), 1H10: enhancing immature virus attachment to endosomal membrane (Wirawan *et al.*^2019^), 5J7: blocking receptor binding and capsid stabilization (Fibriansah *et al.*2015b), E16: blocking capsid reorganization required for virus fusion (Kaufmann *et al.*^2006^), ZIKV-117: capsid stabilization (Hasan *et al.*^2017^), ZKA190: inhibition of either cell attachment or membrane fusion (Wang J *et al.*^2017^), C10: capsid stabilization (Zhang *et al.*^2016^).

**Table 3:** Summary of cryo-EM structures from glycoprotein–antibody complexes.

Genus	Virus name	Antigen	Antibody	Fragment	EMD codes	Resolution (Å)
*Lentivirus*	Human immunodeficiency virus (HIV)	HIV-1 BaL virion	A12	VHH	(Tomo) 5544/5551	
			m36	V_H_	(Tomo) 5552/5553/5554/5555	
			17b	IgG	(Tomo) 5456	22
			VRC01	IgG	(Tomo) 5457	24
			VRC03	IgG	(Tomo) 5458	23
			VRC02	Fab	(Tomo) 5459	23
			VRC02	IgG	(Tomo) 5460	25
			VRC01/17b	IgG	(Tomo) 5461	28
			b12	Fab	(Tomo) 5018/5021	20/20
			17b	Fab	(Tomo) 5020/5023	20/20
			17b/A32	Fab	0466	13.08
		BG505 SOSIP.664	17b/8ANC195	Fab	7516/(Tomo) 3096	3.54/23
			3BNC117	Fab	8644	4.4
			3BNC117/PGT145	Fab	8643	4.3
			3BC315	Fab	3067	9.3
			BG1/8ANC195	Fab	8693	6.2
			PG9/8ANC195	Fab	8695	11.5
			3417	Fab	7552/7553/7554/7555/7556/7557	4.7/4.7/4.7/4.7/4.7/4.7
			VRC34.01	Fab	8125	17
			BF520.1	Fab	9166	4.8
			PGT128	Fab	3121/3120	4.36/4.47
			17b	Fab	8730	8.6
			PGV04	Fab	5779/5780/5781	5.8/7.9/8.2
			PGT151	Fab	9062	4.5
		BG505 DS-SOSIP.664	vFP/VRC03/PGT122	Fab	7622/7621/7459/7460	4/4/3.8/3.6
			vFP	Fab	8420/8421/8422	8.58/14.7/19.6
			PGT145	Fab	8427	6.8
			2G12/VRC03	Fab	8981	8.8
			PGT122/VRC03/FP antibodies	Fab	9189/20189/20191/9359/9320/9319/8977	3.8/4.3/3.5/3.7/4.2/4/3.18
		462c SOSIP.664	VRC01_GL_	Fab	9294/9295/9303/9304	3.8/3.8/4.8/4.8
		B41 SOSIP. 664	17b	Fab	8713	3.7
			PGV04	Fab	8716	7.4
			b12	Fab	8717	3.6
			21c/8ANC195	Fab	9038	4.06
			PGT151	Fab	9030	6.7
		ZM197 SOSIP. 664	VRC01	Fab	3059	9.32
		PC64M18C043 FL Env	PGT151	Fab	7858	3.1
			PGT151/PCT64-35S	Fab	7859	6.8
		PC64M18C043 SOSIP. 664	PGT151	Fab	7860	4.9
		PC64M4C054 SOSIP. 664	PCT64-13C	Fab	7863/7864/7089	5.1/30/13.2
			PCT64-13F	Fab	7862	30
			PCT64-35S	Fab	7865/7866	5.5/8.2
		PC64M4C054 FL Env	PGT151/PCT64-13C	Fab	7861	30
		JR-FL EnvΔCT	PGT151	Fab	3308/3309	4.19/4.3
			PGT151/10E8	Fab	3312	8.8
		AMC011 SOSIP.v4.2	PGV04	Fab	8302	6.2
		KNH1144 SOSIP. gp140	VRC03	Fab	2484	6
			17b	Fab	(Tomo) 5462	8.8
*Lymphocryptovirus*	Epstein-Barr virus (EBV)	glycoprotein	AMMO1	Fab	7344/7345	4.8/10
*Betacoronavirus*	Middle East respiratory syndrome-related coronavirus (MERS-CoV)	S protein	G4	Fab	8783/8784/8785/8786/8787/8788/8789/8790/8791/8792/8793	4/3.6/4.8/4.6/4.8/4.7/5/4.5/4/4/11.5
			LCA60	Fab	0401/0402	3.5/3.6
	Severe acute respiratory syndrome coronavirus (SARS-CoV)	S protein	S230	Fab	0403/0404	4.2/4.5
*Alphainfluenzavirus*	Influenza virus	Influenza virion	6F12	IgG	(Tomo) 6610/6611	25/25
			C179	IgG	(Tomo) 5684/5685	
			7B2	IgG	(Tomo) 6612	25
			3F5	IgG	(Tomo) 6613/6614	25/25
		HA protein	K1915	scFv	8561/8562/8563/8564	4.8/4.8/4.8/4.8
			H7.5	Fab	9142/9143/9145	7.4/9.2/7.4
*Ebolavirus*	Ebola virus (EBOV)	glycoprotein	100/114	Fab	3310/3311	7.2/6.7
			c2G4/c13C6	IgG/Fab	8240	4.3
			c13C6/BDBV91	IgG/Fab	8241	5.5
			c4G7/c13C6	IgG/Fab	8242	4.3
			ADI-15878	Fab	8935/8936	4.14/4.29
		VLPs	c13C6	IgG	(Tomo) 8226	25
			c2G4	IgG	(Tomo) 8227	25
			c4G7	IgG	(Tomo) 8228	25

**Fig. 2:**
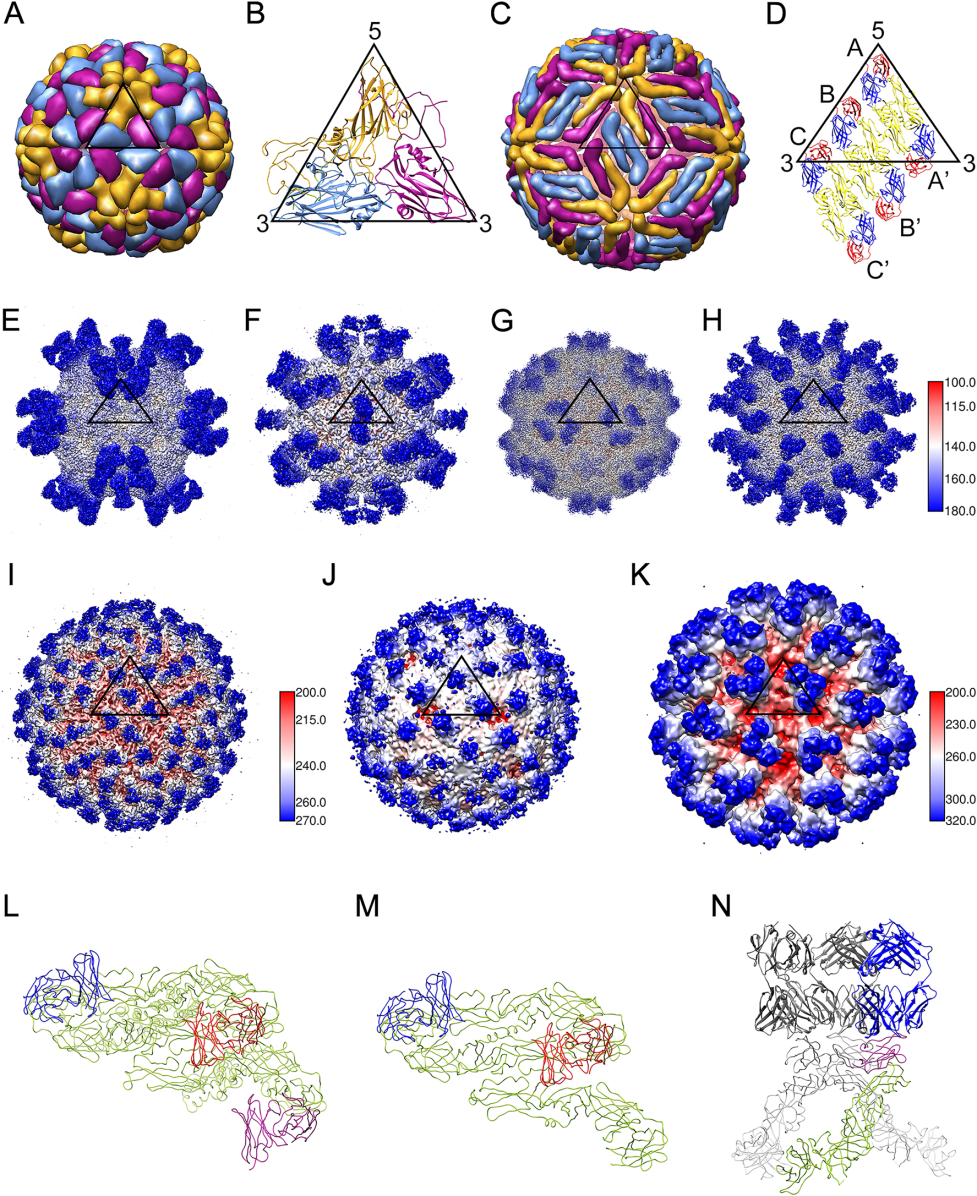
Virus-antibody complexes from picornaviruses (T = pseudo 3) and flaviviruses (T = 3). **A** The capsid shell on human rhinovirus 14 (PDB-4RHV) is formed by 60 copies of VP1 (gold), VP2 (blue), and VP3 (magenta). **B** In an ASU, VP1, VP2 and VP3 are folded with a similar “jelly-roll” topology. **C** The dengue virus capsid structure (PDB-1K4R) is shown as a smooth herringbone lattice of 90 dimers (‘E dimers’). The three E molecules in an ASU are colored gold, blue and magenta. **D** E dimers in two ASUs. The E protein’s ectodomain has three domains: DI (red), DII (yellow) and DIII (blue). **E**–**H** Cryo-EM structures of Fab–picornaviruses complexes, where 60 copies of Fab (blue) bind to the outermost surface of the virus (white) near the 5-, 2-, 3- and q3-fold vertices (5-fold: EMD-6757, 2-fold: EMD-6366, 3-fold: EMD-8762 and q3-fold: EMD-9604, respectively. See Table 1 for related information). An icosahedral ASU is outlined in each map. **I**, **L** 180 copies of Fab 2D22 (blue) bind to DENV2 at 4 °C (EMD-2967). **J**, **M** 120 copies of Fab 2D22 (blue) bind to DENV2 at 37 °C (EMD-2968). **K**, **N** 180 copies of Fab 2H2 bind to immature DENV2 (EMD-5674). There are 60 characteristic spikes on the immature virion. Each spike is a hetero-hexamer consisting of three prM (magenta) and three E molecules (green). Virions are radially colored as in the side bar with the number corresponding to the radius (in Å). Note that the picornavirus and flavivirus particles are not drawn to scale. The external diameters of picornaviruses and flaviviruses are ~ 30 nm and ~ 50 nm, respectively.

Thus far, 3D reconstructions of virus-Fab complexes at atomic resolution are available for some picornaviruses, including rhinovirus B14 (RV-B14) (Dong *et al.*^2017^), human parechovirus 3 (HPeV3) (Domanska *et al.*^2019^), hepatovirus A (HAV) (Cao *et al.*^2019^) and enterovirus D68 (EV-D68) (Zheng *et al.*^2019^) (Table 1). The atomic models of the capsid protein were well-fitted in the cryo-EM density map for HPeV3 (Fig. 1C, 1D). Nevertheless, only the V_H_ and V_L_ domains have clearly defined densities (Figs. 1E, 2G), whereas the density regions in the C_H1_ and C_L_ domains are less ordered, reflecting the flexibility of the linker regions between the constant domains (C_H1_ and C_L_) and variable domains (V_H_ and V_L_). Because the antigen binding site is determined by six hypervariable loops, namely, the complementarity-determining regions (CDRs) on the V_H_ and V_L_ domains, these densities can provide detailed information about the virus-antibody interface. Based on high-resolution structural analysis, CDRs from five HAV antibodies (R10, F4, F6, F7 and F9) interact with a single conserved antigenic site, which has been shown as an attractive target for rational development of antiviral drugs (Wang X *et al.*^2017^; Cao *et al.*^2019^).

## Icosahedral Viruses as Antigens in Cryo-EM Studies

Viruses with icosahedral capsid shells are the most studied antigens in immune complexes by cryo-EM techniques, largely due to their high symmetry. Since perfect symmetry could be broken as observed in many situations, such as symmetry mismatches at the portal vertex of herpesviruses (Parent *et al.*^2018^) and partially mature particles of dengue viruses (Rodenhuis-Zybert *et al.*^2011^), block-based or localized reconstruction strategies are now often applied to icosahedral particles showing conformational flexibility or symmetry mismatches (Ilca *et al.*^2015^; Zhu D *et al.*^2018^). Geometrically, an icosahedral object has 60 equivalent positions related by fivefold, threefold and twofold rotational symmetry. The 60 repeating units occupying each of these positions are referred to as asymmetric units (ASUs). The triangulation number (or T-number) is usually assigned to an icosahedral virus to identify the number of subunits in an ASU. In the simplest T = 1 virus (e.g., parvoviruses), only one capsid molecule is present in the ASU, and the 60 capsid protein copies form an enclosed shell to protect the viral genome. In contrast, in a T = 3 virus (e.g., flaviviruses), the ASU consists of three chemically identical capsid proteins (e.g., the E protein from flaviviruses), each of which undergoes conformational adjustments to occupy three “quasi-equivalent” positions in the ASU. Some icosahedral viruses show “pseudo” T geometry. Picornaviruses (T = pseudo 3) have an ASU of three capsid protein types (VP1, VP2 and VP3), possibly making them a special T = 1 case. Next, we will examine the epitope distribution on these two types of human-infection viruses, and also discuss the binding capacity of Fab molecules on their virions.

Small non-enveloped viruses in the *Picornaviridae* family cause mild or severe human infections, and provide important antigenic particles for cryo-EM studies (Table 1). Crystal structures of rhinovirus (Rossmann *et al.*^1985^) and poliovirus (Hogle *et al.*^1985^) have revealed that on the icosahedral capsid shell, VP1 lies close to fivefold axes and the neighboring VP2 and VP3 are found around threefold axes (Fig. 2A, 2B). Since 1997, outbreaks of hand-foot-and-mouth disease have been increasingly reported, the leading pathogens of which are various enteroviruses, including enterovirus A71 (EV-A71), coxsackievirus A6 (CV-A6), CV-A10 and CV-A16 (Kimmis *et al.*^2018^; Yu and Cowling 2019). Crystallographic and cryo-EM studies have shed some light on the uncoating mechanism of EV-A71, CV-A10 and CV-A16 (Wang *et al.*^2012^; Ren *et al.*^2013^; Zhu L *et al.*2018a). Based on the cryo-EM structures of the enterovirus-Fab complexes, four neutralizing sites have been identified (Fig. 2E–2H) (Zhu R *et al.*^2018^). Site 1 is located near the icosahedral fivefold axis of EV-A71 (MA28-7) (Lee *et al.*^2013^), CV-A6 (1D5) (Xu *et al.*^2017^) and EV-D68 (11G1) (Zheng *et al.*^2019^). While site 2 maps to the VP1 GH-loop across the twofold axis of EV-A71 (22A12 and D5) (Shingler *et al.*^2015^; Ye *et al.*^2016^), site 3 is situated near the threefold axis of EV-A71 (E18, E19 and A9) (Plevka *et al.*^2014^; Zhu L *et al.*2018b) and EV-D68 (15C5) (Zheng *et al.*^2019^). Site 4 is adjacent to the quasi threefold axis of CV-A10 (2G8) (Zhu R *et al.*^2018^).

Some clinically relevant viruses in the *Flaviviridae* family, such as dengue virus (DENV), West Nile virus (WNV), Japanese encephalitis virus (JEV), tick-borne encephalitis virus (TBEV) and Zika virus (ZIKV) (Holbrook 2017; Yang *et al.*^2019^), have been structurally studied as antigenic particles using cryo-EM (Table 2). In contrast with non-enveloped enteroviruses, flaviviruses possess host-derived lipid bilayers with 180 pairs of envelope (E) and membrane (M) proteins on the viral membrane (Perera and Kuhn 2008). Organized as head-to-tail homodimers on the outer surface (Fig. 2C, 2D), the E protein plays important roles in receptor binding and in mediating virus-host membrane fusion. Compared with picornaviruses, flavivirus epitopes do not possess an apparent pattern near symmetrical axes (Fig. 2I, 2J). Based on how Fab interacts with the E dimer, different epitopes on the DENV E protein are classifiable into four groups: (1) those that occur within an E monomer (e.g., 1F4) (Fibriansah *et al.*^2014^); (2) those spanning the adjacent surface of two E molecules from neighboring E dimers (e.g., 14c10) (Teoh *et al.*^2012^); (3) those consisting of amino acid residues from the two E molecules within an E dimer (e.g., 747(4)B7) (Dejnirattisai *et al.*^2015^); and (4) those that occur across three neighboring E molecules (e.g., 5J7) (Fibriansah *et al.*2015b). Largely based on antibody-E complex crystal structures, antibodies may target DI domain, DII fusion loop epitope (FLE) or DIII domain within an E monomer (Dai *et al.*^2016^).

The distance between neighboring epitopes can impact the number of bound antibody molecules on each virion. Although both MA28-7 and 1D5 Fabs bind to site 1 of picornaviruses, only one MA28-7 Fab fragment occupies each fivefold vertex (Lee *et al.*^2013^) while five 1D5 Fab molecules bind each fivefold vertex of CV-A6 (Xu *et al.*^2017^). Compared with 1D5, Fab MA28-7 is closer to the symmetry axis, which renders steric hindrance between possible Fabs, thereby limiting the number of bound Fabs. As another example, the bivalent binding pattern of D5 was characterized in which the two Fab IgG fragments could bind to the GH loops of neighboring VP1 molecules related by twofold symmetry, a finding consistent with the observation that D5 IgG was able to neutralize EV-A71 much more potently than D5 Fab (Ye *et al.*^2016^). Contrastingly, the 22A12 binding sites near twofold axes on EV-A71 are further apart and bivalent binding of an antibody cannot occur (Shingler *et al.*^2015^). In some cases, Fab binding can even change the local arrangement of the E protein to accommodate more Fab molecules. For example, when the total 180 copies of Fab ZKA190 bind to the ZIKV surface, E proteins at the fivefold vertices move apart and steric clash is avoided (Wang J *et al.*^2017^).

Structural variations in the capsid protein at quasi-equivalent positions may also impact the number of bound Fab molecules. Within an ASU, the DENV E protein exists as three conformations showing slight structural variation. 180 copies of Fab 747(4)B7 in total can bind to a DENV virion (Dejnirattisai *et al.*^2015^), suggesting that such variations have no apparent impact on the epitope. However, in other cases, conformational changes can result in a less effective epitope. For example, Fab 1F4 (targeting the DI and DI–DII hinges) does not bind to the E proteins near the threefold vertices where the epitope is partially hidden; consequently, only 120 copies of Fab 1F4 interact with a DENV virion (Fibriansah *et al.*^2014^). Additionally, the capacity of Fab to bind onto a virus particle may not be straightforward when the binding sites are not fully occupied, as shown in the density analysis of ZIKV-117 Fab in a cryo-EM reconstruction (Hasan *et al.*^2017^).

## Immune Complexes for Icosahedral Viruses at Intermediate States

Antibodies have been used to capture intermediate states in the assembly pathway of enteroviruses. There are two major enterovirus particles in infected host cells: empty procapsids (noninfectious) and mature virions (infectious). Upon binding to cellular receptors, the native virions are converted into uncoated intermediates called A(altered)-particles (Shingler *et al.*^2013^). Binding to the E18 antibody transforms infectious EV-A71 into A-particles and triggers genome release (Plevka *et al.*^2014^). Uniquely, CV-A6 A-particles are biochemically and structurally stable, which enabled the A-particle-Fab complex to be reconstructed at a 3.8-Å resolution (Xu *et al.*^2017^). 2G8 shows cross-reactivity against the CV-A10 procapsid, the mature virion, and the A-particle, suggesting that the epitopes on 2G8 are structurally conserved among the three capsid forms (Zhu R *et al.*^2018^).

The dynamic conformational changes occurring during the flavivirus life cycle have also been investigated by cryo-EM. Differing from the mature virus particles with smooth surfaces, immature virions appear as rough particles with 60 spikes comprising three E and three prM molecules (the pr peptide on top of each trimeric spike) (Perera and Kuhn 2008). As the pr peptide is cleaved during virus maturation and is absent in mature virions, anti-prM Fabs (e.g. 2H2 and 1H10) form complexes with immature DENV (Fig. 2K, 2N) (Wang *et al.*^2013^; Wirawan *et al.*^2019^). Highly cross-reactive E53, a fusion-loop-specific antibody, binds preferentially to spikes on immature DENV and WNV particles (Cherrier *et al.*^2009^). Because these antibodies can trap flaviviruses in immature states, the neutralizing mechanism for them may depend on their capacity to block the normal transition occurring during the maturation process.

Many cryo-EM studies have been performed to investigate antibody-virus particle interactions for different functional states, including the following ones: (1) Intermediate complexes during ‘breathing’ motion. Although each cryo-EM reconstruction usually represents a static snapshot of a specific conformation, structural plasticity in immune complexes can also be visualized. Fab 1A1D-2 induces large conformation changes in the E protein and binds to normally partially hidden epitopes (Lok *et al.*^2008^). Cryo-EM studies have also revealed that Fab 1A1D-2 only binds to epitopes near the fivefold and threefold vertices, suggesting that the extent of the breathing might be not evenly distributed over the viral surface. (2) Size variations in the viral shells at different temperatures. It was found that when exposed to 37 °C, DENV virions expand in size when compared with the structure at 4 °C (Fibriansah *et al.*^2013^). The unexpanded virions at 4 °C are covered by 180 copies of the 2D22 Fab (Fig. 2I, 2L), whereas only 120 Fab copies are present on some expanded virions at 37 °C (Fig. 2J, 2M), as based on 2D and 3D classification of extracted virus-antibody particles (Fibriansah *et al.*2015a). (3) Fusion intermediates. A low pH-triggered rearrangement of the E protein is required for virus–cell membrane fusion during entry of flaviviruses into the cell. The E16 Fab trapped WNV in a prefusion state when the virions were exposed to low pH (Kaufmann *et al.*^2009^). C10, a bNAb for DENV, can structurally lock the E protein of ZIKV at acidic conditions (Rouvinski *et al.*^2015^; Zhang *et al.*^2016^).

## Enveloped Viruses Without Icosahedral Symmetry

Many severe human diseases are caused by structurally polymorphic enveloped viruses (e.g., HIV-1, influenza and Ebola viruses). Glycoprotein-specific antibody-inducing epitopes on the viral surface have been studied directly using cryo-ET. The open conformations of the HIV-1 Env spike induced by Fab b12 or CD4/Fab 17b have been characterized using cryo-ET analysis (Liu *et al.*^2008^). The extent to which the C179 antibody bound to the stem domain of hemagglutinin (HA) on the influenza virus was also investigated by cryo-ET, revealing that most of the HA trimers on virions were accessible to this antibody (Harris *et al.*^2013^).

High-resolution SPA of glycoprotein–antibody complexes requires stabilized protein samples, a good example of which is the engineered HIV-1 Env trimer with SOSIP mutations (Sanders *et al.*^2002^). Mature HIV-1 possesses trimers of gp41/gp120 homodimers as the surface spikes. Although some spike epitopes are present on the gp120 monomer, it would be desirable to choose native-like trimers for structural analysis because they are the major target of the neutralizing antibodies elicited by natural infection (Sanders and Moore 2017; Ward and Wilson 2017). SOSIP mutants contain an introduced disulfide (SOS) bond between the gp120 and gp41 ectodomains, and an introduced isoleucine to proline mutation in gp41 to promote trimer formation. Engineered glycoprotein trimers from other enveloped viruses (e.g., Middle East respiratory syndrome coronavirus, MERS-CoV, and parainfluenza virus types 1–4) were designed to present the antigenically optimal prefusion conformation (Pallesen *et al.*^2017^; Stewart-Jones *et al.*^2018^). Glycoproteins from Ebola virus were modified by removing the mucin-like domain, assembled as soluble trimers, and then studied in a complex with the ADI-15878 Fab by SPA (Murin *et al.*^2018^). Except for the above-mentioned trimeric forms, glycoprotein complexes containing more than one viral glycoprotein (e.g., gH/gL/gp42 from Epstein-Barr virus) have also been used for cryo-EM antibody–antigen studies (Snijder *et al.*^2018^).

Cryo-EM structures of glycoprotein–antibody complexes are usually captured in intermediate states. Twenty antibody classes targeting six epitopes on the prefusion closed HIV-1 Env trimer have been characterized using SPA (Table 3) and crystallographic studies (Chuang *et al.*^2019^). ‘Breathing’ by HIV-1 B41 SOSIP.664 trimers was found to expose the b12 epitope (Ozorowski *et al.*^2017^), and a similar motion by influenza HA protomers was also revealed by SPA (Turner *et al.*^2019^). S230 binding induces fusogenic conformational rearrangements in the SARS-CoV S glycoprotein, while the MERS-CoV S glycoprotein remains its prefusion conformation upon LCA60 binding (Yuan *et al.*^2017^; Walls *et al.*^2019^).

## Virus-Like Particles (VLPs) as Antigen Presentation Platforms

VLPs with features that are structurally and immunologically indistinguishable from live viruses are used as alternative models for cryo-EM studies, especially when the viruses need to be manipulated in high-level biosafety facilities. Chikungunya virus (CHIKV) is a mosquito-transmitted human pathogen with T = 4 icosahedral symmetry. On its surface, three E2 molecules form the major component of the viral spike and serve as the main target for antibodies. Because handling live infectious CHIKV requires biosafety level 3 facilities, cryo-EM studies are performed with the safe CHIKV vaccine strain (CHIKV 181/25) (Fox *et al.*^2015^) or CHIK VLPs (Akahata *et al.*^2010^; Sun *et al.*^2013^; Jin *et al.*^2015^). The CHK-265 Fab cross-links two E2 molecules from neighboring spikes (Fox *et al.*^2015^), while the footprints of C9 and IM-CKV063 Fabs on VLPs span the neighboring E2 subunits within one viral spike (Jin *et al.*^2015^). CHK-152 may cross-link the flexible domain B to the domain A within an E2 molecule, and thus inhibiting the exposure of the fusion loop on domain II of E1 (Sun *et al.*^2013^).

VLPs have also served as controllable scaffolds for loading antigenic cargos (Charlton Hume and Lua 2017). Currently, the most commonly used VLPs are rigid icosahedral or helical particles, and flexible platforms are beginning to be promising roles for antigen loading (Hudalla *et al.*^2014^; Rao *et al.*^2018^). To protect against different human papillomavirus (HPV, pseudo T = 7 icosahedral) infections, chimeric VLPs containing the epitopes from three HPV types have been generated and studied by cryo-EM (Li *et al.*^2018^). Recently, computationally designed nanoparticles have also been examined by cryo-EM as a new platform for presenting the respiratory syncytial virus F glycoprotein trimer (Marcandalli *et al.*^2019^). Being able to add antigenic protein modules to tailorable platforms is a tantalizing way of studying highly virulent viruses like Crimean-Congo hemorrhagic fever, Nipah, and Ebola viruses. It is also likely that the cryo-EM characterization of VLPs and nanoparticles will accelerate the development of new vaccine platforms.

## Conclusions

Cryo-EM has evolved in recent years into a powerful technique for elucidating the structural basis of virus-antibody interactions. Compared with traditional X-ray crystallography, cryo-EM offers the following advantages: (1) it can investigate conformational epitopes with sequentially discontinuous residues on icosahedral virions; (2) it avoids tedious screening for diffractable crystals and can be incorporated into a standardized process for rapid and rational vaccine development; (3) it can help with analyzing intrinsic heterogeneous samples, like highly glycosylated viral glycoproteins; and (4) it can be exploited for developing and characterizing high-quality vaccine platforms. Finally, cryo-EM studies of antigen–antibody complexes are beginning to clarify the mechanisms of epitope–paratope recognition at atomic resolution, so we expect that high-resolution cryo-EM structures will play more important roles in future at guiding vaccine development.
